# A Functional Genome-Wide *In Vivo* Screen Identifies New Regulators of Signalling Pathways during Early *Xenopus* Embryogenesis

**DOI:** 10.1371/journal.pone.0079469

**Published:** 2013-11-14

**Authors:** Siwei Zhang, Jingjing Li, Robert Lea, Enrique Amaya, Karel Dorey

**Affiliations:** The Healing Foundation Centre, Faculty of Life Sciences, University of Manchester, Manchester, United Kingdom; University of Colorado, Boulder, United States of America

## Abstract

Embryonic development requires exquisite regulation of several essential processes, such as patterning of tissues and organs, cell fate decisions, and morphogenesis. Intriguingly, these diverse processes are controlled by only a handful of signalling pathways, and mis-regulation in one or more of these pathways may result in a variety of congenital defects and diseases. Consequently, investigating how these signalling pathways are regulated at the molecular level is essential to understanding the mechanisms underlying vertebrate embryogenesis, as well as developing treatments for human diseases. Here, we designed and performed a large-scale gain-of-function screen in *Xenopus* embryos aimed at identifying new regulators of MAPK/Erk, PI3K/Akt, BMP, and TGF-β/Nodal signalling pathways. Our gain-of-function screen is based on the identification of gene products that alter the phosphorylation state of key signalling molecules, which report the activation state of the pathways. In total, we have identified 20 new molecules that regulate the activity of one or more signalling pathways during early *Xenopus* development. This is the first time that such a functional screen has been performed, and the findings pave the way toward a more comprehensive understanding of the molecular mechanisms regulating the activity of important signalling pathways under normal and pathological conditions.

## Introduction

During embryonic development, cells constantly receive and emit signals that determine their position, fate and migratory behaviour [1]–[3]. The vast variety of developmental decisions are made using a relatively small number of signalling pathways, such as the Hedgehog, Wnt, Transforming Growth Factor-β (TGF-β), Bone Morphogenic Protein (BMP), Receptor Tyrosine Kinase (RTK), Notch, JAK/STAT and nuclear hormone pathways [4]. It is the precise regulation of these pathways together with cross talk between them that ensure an accurate biological output. De-regulation of any of these signalling pathways is often associated with developmental defects and diseases [5], [6]. Therefore, identifying molecules that regulate these pathways under physiological conditions is an important prerequisite to understand how mis-regulation of these pathways leads to abnormal development and disease.

Several gain and loss-of-function genetic screens have been performed in order to identify novel regulators of growth factors signalling during development. This approach has been particularly successful in *Drosophila*, where both gain and loss of function screens are possible [7], [8]. For example, an overexpression screen in *Drosophila* led to the identification of many genes influencing FGF signalling [9]. Amongst vertebrates, *Xenopus* embryos provide a powerful system to investigate the role of growth factor signalling. Indeed this system has been instrumental in establishing much about what we know about the importance of various signalling pathways during early embryogenesis [3], [10]–[12]. For example, a role of FGF signalling during early vertebrate development was first shown in *Xenopus* [3], [13]–[15], and the importance of Wnt, TGF-β/Nodal/Activin, and BMP signalling during early embryogenesis was also uncovered by early studies of *Xenopus* development [16]–[19]. *Xenopus* has also proven to be a very useful model in identifying novel factors that are important for early embryonic development [20], [21]. But until now, screens for developmental regulators have been mostly based on phenotypes, which have the disadvantage that they cannot distinguish primary from secondary effects. To overcome this, we designed and performed a screen, which provides a more immediate readout based on the biochemical assessment of the activation state of several intracellular signalling pathways.

Here, we first characterised a set of antibodies to monitor the activation state of several signalling pathways, including the TGF-β/Nodal, BMP, MAPK/Erk, and PI3K/Akt pathways, which allowed us to analyse the temporal dynamics of these signalling pathways during early stages of *Xenopus* development. We then performed an *in vivo* large-scale gain-of-function screen aimed at identifying new molecules able to modulate the activity of the TGF-β/Nodal, BMP, PI3K/Akt, and FGF pathways during early vertebrate embryogenesis. Overall, 20 potential regulators have been identified out of 2,880 screened full-length clones isolated from egg, gastrula, and neurula stages. Finally, *in situ* hybridisation analyses have revealed that half of the putative modulators of growth factor signalling are regulated at the transcriptional level in time and space. Together, these results open new avenues of investigation in better understanding the regulation of signalling pathways during embryonic development.

## Materials and Methods

### Ethics Statement

All animal experiments were approved from the University of Manchester Animal Welfare Centre and were covered by a UK Home Office Project Licence.

### Preparation of mRNA pools for microinjection

We used of the *X. tropicalis* full-length cDNA library (known as xt3: fl2) for the large-scale gain-of-function screen [21], [22]. This full-length library can be obtained from Source BioScience (http://www.lifesciences.sourcebioscience.com). Briefly, each 96-well plate was subdivided into 12 pools by column, each containing 8 clones. The clones were individually cultured in 96-well deep plates and pooled for plasmid extraction. Plasmids were linearised using AscI, and capped mRNAs were synthesized *in vitro* using SP6 RNA polymerase (NEB). The quality of synthesised mRNA pools was checked by 1% agarose gel electrophoresis.

### Injection, collection, and extraction of *X. laevis* embryos

*Xenopus laevis* eggs were artificially fertilised to ensure synchronized development. Embryos were treated from stage 6.5 until stage 10.5 with the indicated chemical inhibitors at the following concentrations: FGF Receptor inhibitor SU5402 (Sigma): 40 µM; PI3K inhibitor LY294002 (Cell Signaling): 50 µM; TGF-β Receptor inhibitor SB505124 (Sigma-Aldrich): 20 µM. DMSO was added at a final concentration of 1% (v/v), representing the highest concentration used as solvent for the inhibitors. For RNA pools, a total of 6.4 ng of mRNA was injected into each *X. laevis* embryo at the 1–2 cell stage. For subsequent de-convolution of single clones from pools, 800 pg of mRNA was injected. Treated/injected embryos were collected at stage 8, 10.5 and 14 from each pool. For each stage, 7 embryos were collected. Collected embryos were homogenized using PK buffer to extract proteins and the yolk was eliminated by centrifugation [23]. Cleared supernatant were denatured using Laemmli sample buffer for subsequent SDS-PAGE and Western blot analysis.

### SDS-PAGE and Western blot detection

The equivalent of 1 embryo lysate was loaded onto 8% SDS-PAGE and after electrophoresis, proteins were transferred onto PVDF membrane. The following primary antibodies were used: anti-phospho-Akt S473 (Cell Signaling, #4051); anti-phospho-Erk 1/2 T180/Y182 (Sigma-Aldrich #E7028), anti-phospho-Smad1/5/8 (Cell Signaling #9511); anti-phospho-Smad2 A5S (Millipore #05-093); anti-phospho-LRP6 (Cell Signaling #2568), anti-Erk (Cell Signaling #9102); anti-Akt (Cell Signaling #4691); anti-Smad2 (BD Biosciences #610842); and anti-α-tubulin (Sigma Aldrich #T9026). All antibodies were used at 1∶1000 concentration except for anti-phospho-Erk 1/2 (1∶10,000) and anti-α-tubulin (1∶100,000). Tris-buffered saline with 0.1% Tween 20 (TBST) and 5% milk was used for blocking, except for the anti-phospho-Smad1/5/8 where TBST with 5% BSA was used. Chemiluminescence detection was performed using HRP-conjugated anti-rabbit (1∶40,000; Dako #P044801) or anti-mouse (1∶100,000; Dako # P044701) antibody combined with Millipore Immobilon ECL reagent (#WBKLS0500).

### In situ hybridisation

Whole-mount *in situ* hybridisation was performed using *X. tropicalis* embryos as described [24]. Expressed Sequence Tags (EST) constructs were linearised by either EcoRI or ClaI as appropriate, followed by *in vitro* transcription using T7 RNA polymerase (Roche) and digoxigenin RNA labelling mix (Roche). Chromogenic detection was performed using BM purple AP substrate (Roche).

## Results and Discussion

### Characterisation of phospho-specific antibodies

**Figure 1:**
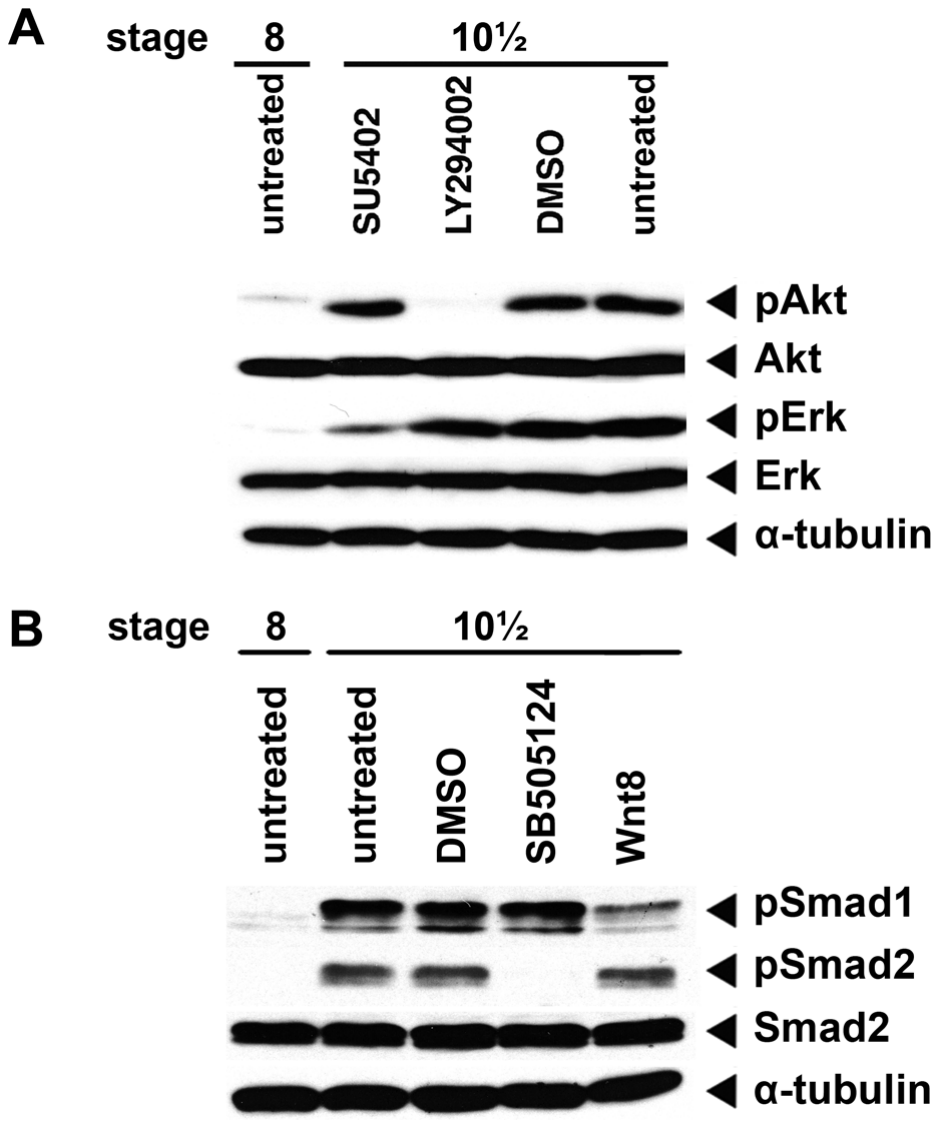
Characterisation of phospho-specific antibodies. (A) Characterisation of anti-phospho-Akt (pAkt) and anti-phospho-Erk (pErk) antibodies, the PI3K/Akt inhibitor LY294002 and FGF inhibitor SU5402 were used to inhibit Akt and Erk phosphorylation in gastrula embryos, respectively. 1% DMSO was used to exclude any possible interference from the inhibitor solvent. Anti-Erk (Erk), anti-Akt (Akt) and anti-α-tubulin (α-tubulin) were used as loading controls. (B) Characterisation of anti-phospho-Smad1/5/8 (pSmad1) and anti-phospho-Smad2 (pSmad2) antibodies. The TGF-βRI inhibitor SB505124 was used to inhibit Smad2 phosphorylation in gastrula embryos; injection of *wnt8a* mRNA was used to inhibit *bmp4* expression, thus preventing Smad1/5/8 phosphorylation. All inhibitors have been added at stage 6. 1% DMSO was used to exclude any possible interference from the inhibitor solvent. Smad2 and α-tubulin serves as internal controls to ensure equal loading in all lanes.

We first identified a set of phospho-specific antibodies, which permit the monitoring of the activation state of several signalling pathways known to play major roles during early *Xenopus* development, including the BMP, TGF-β/Nodal, MAPK/Erk, and PI3K/Akt signalling pathways. Based on the literature, we tested an anti-phospho-Smad1/5/8 (pSmad1, downstream of BMPs) and an anti-phospho-Erk antibody (pErk, downstream of RTKs) [23]. We also tested an antibody recognising phospho-Akt (pAkt S473 from Cell Signalling) downstream of active PI3K and a new anti-phospho-Smad2 antibody (clone A5S from Millipore), downstream of active TGF-β/Nodal signalling. To confirm the specificity of these antibodies, we performed Western blot analyses using protein extracts from mid-blastula stage embryos (stage 8), when most signalling pathways are inactive, and early gastrula stage embryos (stage 10.5) when most signalling pathways are active (Figure 1). Our experiments confirmed that phospho-Akt (pAkt), phospho-Erk (pErk), phospho-Smad1/5/8 (pSmad1), and phospho-Smad2 (pSmad2) are low or absent in stage 8 embryos, but are abundant in stage 10.5 embryos (Figure 1A–B). To test further the specificity of the antibodies, we treated embryos with the FGFR inhibitor (SU5402) to decrease pErk levels or the PI3K inhibitor LY294002, which would be expected to decrease pAkt levels in gastrula stage embryos [25], [26]. Indeed, we observed a significant reduction in pErk levels in stage 10.5 embryos treated with SU5402 and a decrease in pAkt levels in stage 10.5 embryos treated with LY294002 (Figure 1A). Intriguingly, we did not find a change on pAkt levels in embryos treated with SU5402, suggesting that PI3K activity at this stage of development is not dependent on FGFR signalling (Figure 1A). Pre-treatment of stage 8 embryos with the TGF-β Receptor inhibitor, SB505124, prevented Smad2 phosphorylation (Figure 1B). Injection of *wnt8* mRNA at the 1-cell stage, which is known to inhibit *bmp4* expression [27], resulted in a significant decrease in Smad1 phosphorylation (Figure 1B).

### Time course of activity on different signalling pathways

**Figure 2:**
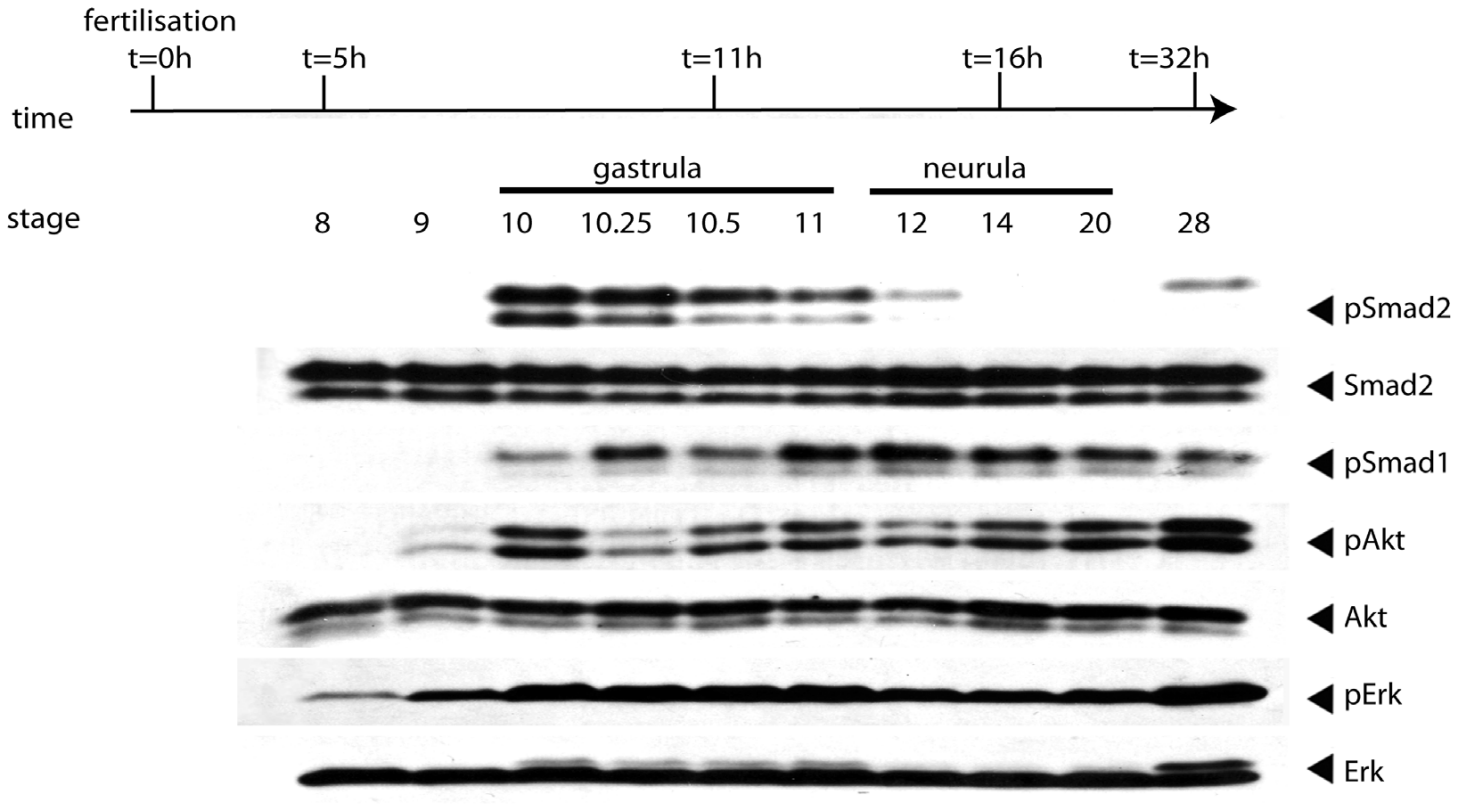
Kinetics of the activation of signalling molecules during early *Xenopus* development. *X. laevis* embryos were collected at the time indicated and subjected to Western blot analysis. Membranes were probed with anti-phospho-Smad1/5/8 (pSmad1) antibody for monitoring BMP activity, anti-phospho-Smad2 (pSmad2) antibody for TGF-β/Nodal signalling, anti-phospho-Erk (pErk) for MAPK/Erk signalling and anti-phospho-Akt (pAkt) for PI3K/Akt signalling. Anti- Smad2, anti- Akt, and anti-Erk were used as loading controls to ensure all lanes have been loaded equally.

Having defined a set of antibodies allowing us to monitor the BMP, TGF-β/Nodal, MAPK/Erk, and PI3K/Akt signalling, we performed a time-course experiment to define the temporal dynamics of the activation state of these signalling pathways during early development. To this end, we collected embryos at stage 8 and 9 (blastula stages), 10, 10.25, 10.5, and 11 (gastrula stages), 12, 14, 20 (neurula stages) and 28 (early tadpole stage). After protein extraction, samples were analysed by Western blot assays to determine the phosphorylation status of the signalling molecules previously described (Figure 2). In accordance with previous studies, most signalling pathways were silent or had very low activity at the blastula stages (Figure 2) [28]. As gastrulation began (stage 10), Smad1/5/8, Smad2, Akt, and Erk became phosphorylated or their phosphorylation state increased (Figure 2). At the end of gastrulation, the phosphorylation levels of Smad2 and Erk decreased whilst phosphorylated Smad1/5/8 and Akt remained elevated. Smad2 and Erk phosphorylation increased again by the early tadpole stage (stage 28). This time-course analysis indicates that different signalling pathways have very precise kinetics of activation and de-activation, suggesting that they are tightly regulated during embryonic development.

### Overview of the in vivo screen strategy and proof of principle pilot screen

**Figure 3:**
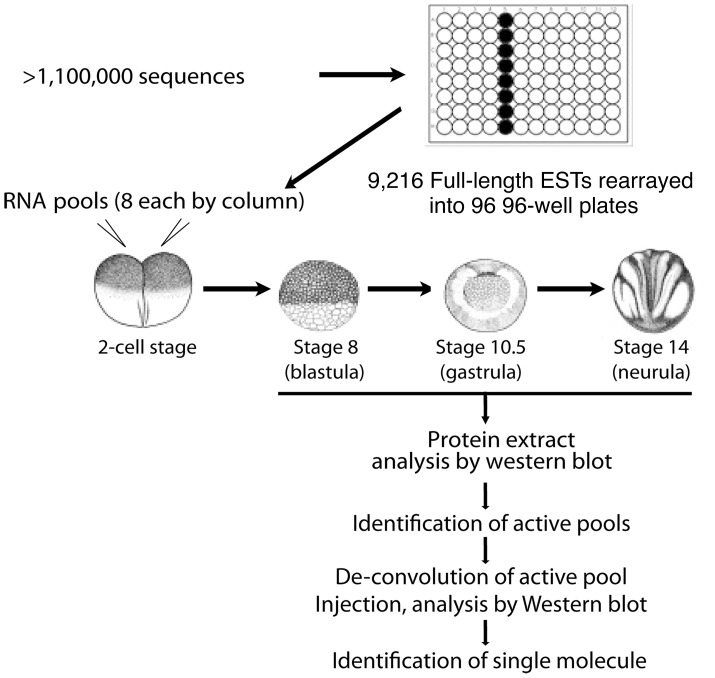
Flowchart of the experimental procedure of the screen. A *X. tropicalis* library of unique, full-length clones has been established based on sequence comparison and clustering of over 1,220,000 ESTs, and rearrayed in a 96-well plate format. Pools of 8 mRNAs were prepared from pooled bacteria culture and *in vitro* transcription. Then *in vitro* transcribed mRNA pools were injected into fertilized *X. laevis* embryos at 1–2 cell stage. After microinjection, injected embryos were collected at stage 8 (blastula), stage 10.5 (gastrula), and stage 14 (neurula). Protein extracts from embryos were loaded onto SDS-PAGE for subsequent Western blot analysis. Antibodies used include anti-phospho-Smad1/5/8, anti-phospho-Smad2, anti-phospho-Akt, and anti-phospho-Erk. Once a potential active pool was identified, the pool was de-convoluted and single molecule injection was performed to identify the active molecule.

The time-course data of the various signalling pathways motivated us to design a large-scale gain-of-function screen aimed at identifying new modulators of the pathways during early embryonic development (Figure 3). For the screen we decided focus on three stages of development: the early blastula stage (stage 8) when only phospho-Erk is detectable, albeit at low levels; the mid-gastrula stage (stage 10.5) when all the signalling molecules analysed were phosphorylated; and the neurula stage (stage 14) when some signalling pathways remained active (BMP and PI3K/Akt), while Erk activity was reduced and Nodal signalling became undetectable. Using these three stages, we have a unique opportunity to screen for molecules able to perturb the natural activation and de-activation states of multiple pathways without the need of stimulating cells with non-physiological amounts of growth factors. Furthermore, since the screen is performed in whole embryos, as opposed to performing it on cell lines, the chance of identifying molecules important during early development would be increased. Indeed, a long-standing goal in developmental biology has been to understand how signalling pathways are precisely regulated to control cell fate decisions and coordinate cell movements, and we expected that our designed strategy would be able to provide a means to identify novel molecules that modulate the various signalling pathways during early development. Finally, since the assay directly monitors the activation state of the signalling pathways, rather than their downstream phenotypic effects, we expected that it might permit a more immediate and specific means of identifying direct modulators of the various pathways.

In brief, the general approach of the gain of function screen was to generate *in vitro* transcribed mRNA in pools of eight from our full-length EST library, inject these pools into one to two stage embryos, and then identify pools that alter the phosphorylation state of phosphorylation state of any of the key signalling pathways via Western blot analyses. Once active pools have been identified, we would proceed by de-convoluting them in order to identify the single active clones contained within the positive pools (Figure 3).

**Figure 4:**
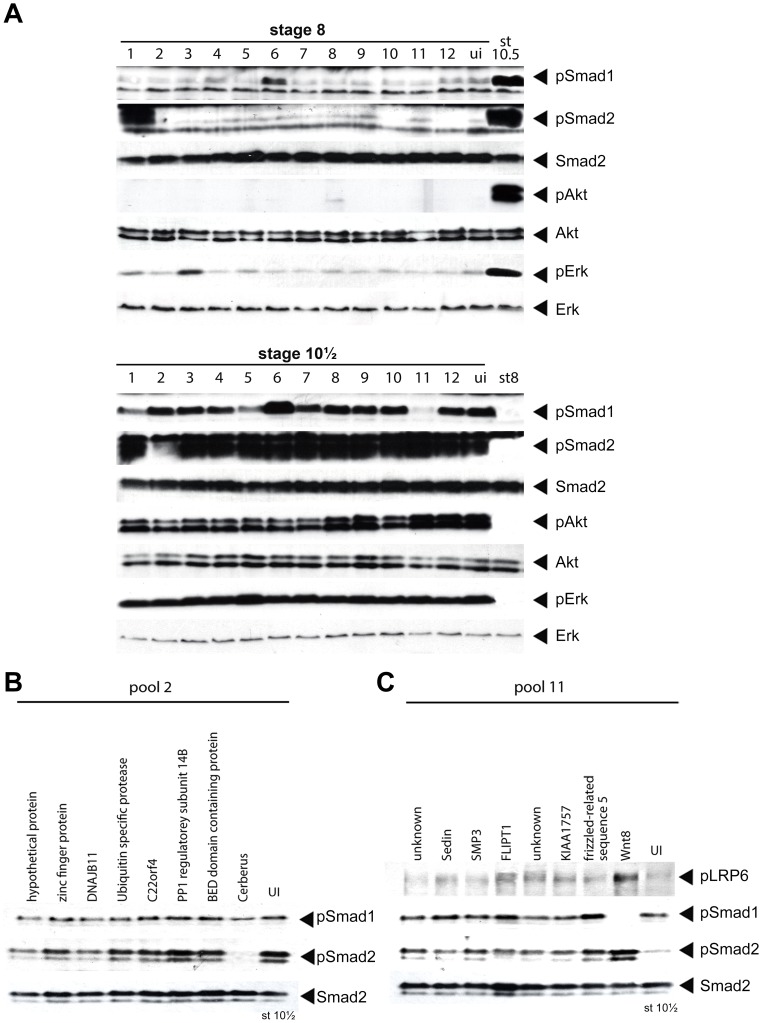
Proof of principle of the screen. (A) 12 pools, each one with one clone of known activity, were selected from the full-length EST library and injected into embryos as described. Protein extracts from collected embryos were subjected to Western blot using indicated antibodies to observe phosphorylation changes of specific signalling molecules at blastula and gastrula stages. Note the reduction of phospho-Smad2 activity at gastrula stage on pool 2, and reduction of phospho-Smad1/5/8 activity on pool 11. (B) De-convolution of pool 2. *cerberus* is identified as a negative regulator of Smad2 (pSmad2, lower panel) but not of Smad1 (pSmad1, upper panel) phosphorylation at gastrula stage. (C) De-convolution of pool 11. *wnt8a* is identified as a negative regulator of Smad1/5/8 phosphorylation (pSmad1, middle panel) and activator of Wnt signalling (pLRP6, upper panel) at gastrula stage. UI: uninjected.

Since a screen using phospho-specific antibodies as a means of assessing the activation state of signalling pathways had not been attempted previously, we first decided to perform a pilot screen to demonstrate its feasibility. To this end, we selected 10 clones with known activities from our full-length EST library (Figure S1 and Table 1) [21], [22]. In addition, we also introduced 2 clones identified in a previous screen, which caused gastrulation defects [21], namely *FGFR1 oncogene partner* (*fgfr1op*, pool 10) and a putative metalloprotease similar to hatching gland-like XheI protein (pool 12), to determine whether these two clones could alter the phosphorylation state of the signalling molecules in our screen. To simulate the same conditions of the full screen, we rearrayed the 12 clones together with their 7 corresponding neighbouring clones from their respective columns onto a new 96-well plate (Figure S1). For example, the clone corresponding to *nodal-related 1* (also known as *xnr1*), TGas124h10, is on plate 044 at position H04 in the full-length clone library (information available at http://genomics.nimr.mrc.ac.uk). We therefore picked the eight clones in the “A04” column (from position A04 to H04) from plate 044, re-arrayed them into one column of a new 96-well plate, and extracted plasmid from these eight clones to make a single test pool. The same general strategy was used to generate the remaining eleven test pools used in the pilot screen (Figure S1). Plasmid linearization and mRNA synthesis were then performed for the twelve test pools. 6.4 ng of mRNA per pool was injected into the marginal zone of 1–2 cell stage embryos and the injected embryos were allowed to develop to the mid-blastula (stage 8), mid-gastrula (stage 10.5) and neurula stage (stage 14) before they were collected. Embryos were then processed for Western blot analyses to assess the phosphorylation state of key signalling molecules in the Smad1/5/8, Smad2, Akt, and Erk pathways (Figure 4A and data not shown).

**Table 1:** Selection of the clones used to validate the screen strategy.

Pool	Clone ID	Gene name	Known effects	Effect(s) on signalling pathways as detected during the screen
1	TGas124h10	*xnr1*	Nodal activator	Smad2 activation (stage 8)/Smad1 inhibition (stage 10.5)
2	TGas102k04	*cerberus*	BMP, Nodal, and Wnt inhibitor	Smad2 inhibition at stage 10.5
3	TNeu076b03	*fgfr1*	MAPK activator	Erk activtion at stage 8
4	TEgg022o22	*mkp1*	JNK inhibitor	none
5	TNeu122a14	*noggin2*	BMP inhibitor	Smad1 inhibition at stage 10.5
6	TEgg062o06	*bmp2*	BMP activator	Smad1 activation at stage 8
7	TTbA021m16	*pten*	Akt inhibitor	none
8	TEgg047o09	*bambi*	BMP and Nodal inhibitor	none
9	TEgg078l20	*tob*	BMP inhibitor	none
10	TGas107e20	*fgfr1op*	gastrulation defect	none
11	TNeu118d19	*wnt8a*	Wnt activator	Smad1 inhibition and Wnt activation at stage 10.5
12	TNeu108l05	*metalloprotease*	gastrulation defect	none

Out of the 12 pools, 6 showed changes in the phosphorylation state of the signalling molecules tested (Table 1 and Figure 4A). Notably, only 3 pools out of the 12 screened (pool 7, 8, and 9) failed to produce the expected results based on the published literature (Figure 4A). This may be attributed to several reasons. Firstly, the amount of mRNA may not have been sufficient to reach an effective dose. Secondly, the injected molecule may regulate the expected signalling pathway through mechanism other than changing the phosphorylation state of signalling molecules assayed in the screen. Finally, most clones from the EST library harbour 5′ and 3′ untranslated regions (UTRs) that may hamper the translation efficiency of their mRNAs. However, it is important to note that the screen itself is highly specific since none of the pools gave false positives. We did not find significant changes with the two clones identified from previous screen results (pool 10 and 12), suggesting that they either function in parallel or downstream of the signalling molecules tested in our screen.

We then decided to de-convolute two pools to determine whether we could isolate the active clones. For pool number 2, we were able to confirm that the active clone corresponded to *cerberus* (TGas102k04) [29]. Cerberus is a secreted protein expressed in the anterior domain of the mesendoderm [30]. It is thought that the ability of Cerberus to inhibit Nodal, BMP, and Wnt signalling is essential for the induction of the head structure in *Xenopus* [31]. As expected, we found that over-expression of *cerberus* strongly inhibited Smad2 phosphorylation but surprisingly had no effect on Smad1 phosphorylation (Figure 4A, 4B). This is the first time the effect of *cerberus* overexpression on the phosphorylation state of the downstream signalling effectors has been tested directly. It raises the possibility that the molecular mechanism of Cerberus activity is more complex than just preventing BMP and Nodal ligands to bind their receptors [31]. For pool number 11, *wnt8a* (TNeu118d19) was identified as the sole regulator that had a dual effect of inhibiting Smad1/5/8 and activating Smad2 (Figure 4C) [27]. We also assessed the phosphorylation state of the Low-density lipoprotein receptor-related protein 6 (Lrp6), a Wnt co-receptor, which is phosphorylated when canonical Wnt signalling is active (Figure 4C). As expected, the phosphorylation level of Lrp6 was increased in *wnt8a* over-expressed embryos, which indicated the canonical Wnt signalling was activated. Taken together, the pilot screen validated our experimental approach.

### Result of the screen

**Figure 5:**
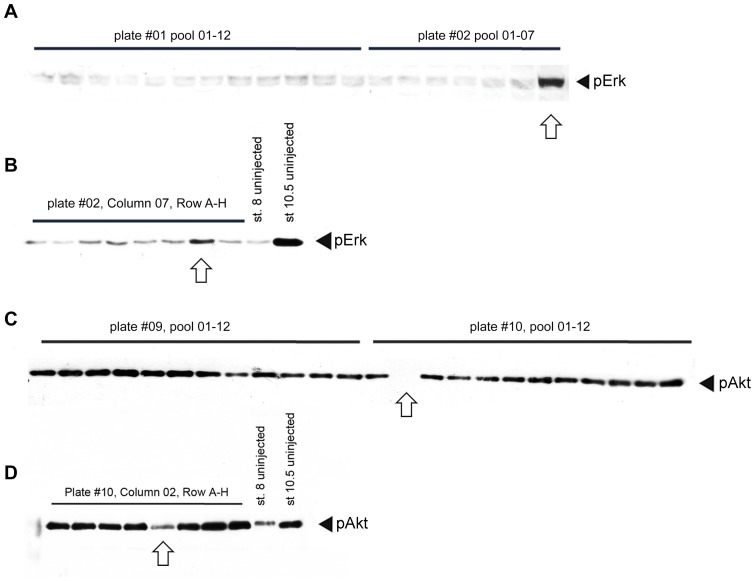
Examples on identification and de-convolution of active regulators. (A–B) identification of the MAPK/Erk activator *fbxo43* (*erp1*). (A) Western blot of stage 8 embryos injected with 12 pools (01–12) derived from plate #01 and 7 pools (01–07) from plate #02 and probed with anti-phospho-Erk (pErk) antibody. The arrow indicates increased Erk phosphorylation upon injection of mRNAs derived from plate #02, pool 07. (B) De-convolution of the above pool. Embryos injected with single RNAs were collected at stage 8 and uninjected lysate from stage 8 and stage 10.5 were used as negative and positive control of Erk phosphorylation respectively. The arrow indicates the active clone of TEgg009F05, identified in plate #2, column 08, row G. This clone was confirmed as the *X. tropicalis fbxo43* (*erp1*) gene. (C–D) Identification of PI3K/Akt inhibitor *prkaca*. (C) Western blot of stage 10.5 embryos injected with 24 pools (01–12) derived from plate #09 and #10 and probed with anti-phospho-Akt (pAkt) antibody. The arrow indicates decreased Akt phosphorylation upon injection of mRNAs derived from plate #10, pool 02. (D) De-convolution of the above pool. mRNA synthesis, injection, and Western blot were performed as in (B) except that stage 10.5 embryos were used. The arrow indicates the active clone of TEgg046d13 is identified in plate #09, column 02, row E. This clone was later identified as encoding the *X. tropicalis prkaca* gene.

**Figure 6:**
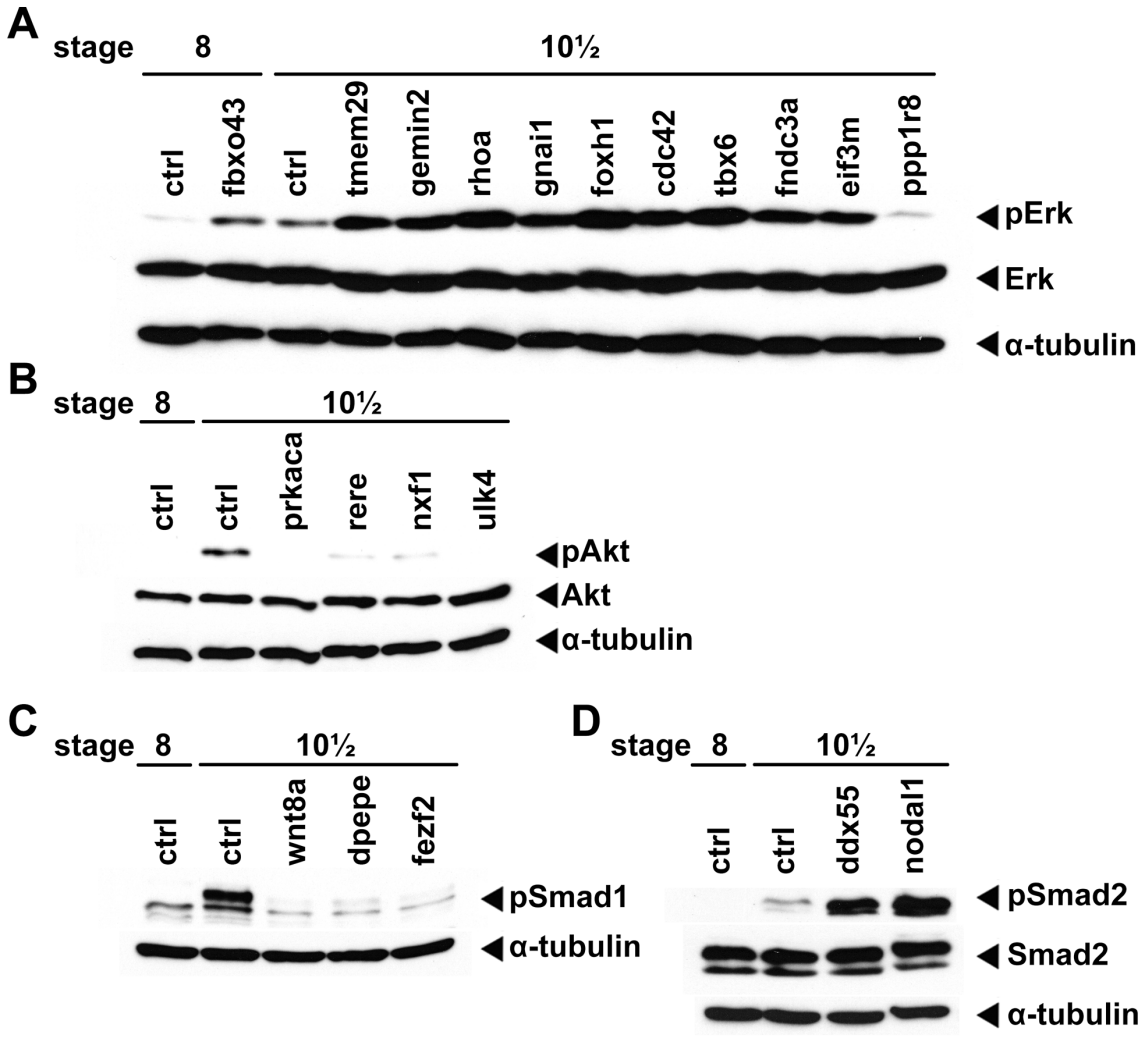
Results of the screen. The 20 active clones identified during the screen were individually injected and analysed by Western blot to demonstrate their abilities to modulate the activities of different signalling pathways as shown in Table 3. Embryos were collected at the indicated stages, processed, and analysed by Western blot to assess the activities of Erk (pErk, panel A), Akt (pAkt, panel B), BMP (pSmad1, panel C), and TGFβ/Nodal (pSmad2, panel D). Control (ctrl) denotes uninjected embryos. Anti-Erk (Erk), anti-Akt (Akt), anti-Smad2 (Smad2) and anti-α-Tubulin (α-Tubulin) were used as loading controls.

Having demonstrated the feasibility of our strategy, we then performed a screen on approximately a third of our full-length library (2880 of the 9216 clones) [21], [22]. Since the full-length clone library was already in a 96-well plate format, we used 16 plates containing clones isolated from egg stage library (TEgg series, plates 01–16), 12 plates containing clones from gastrula stage library (TGas series, plates 33–38, 41–44, 47–48), and 2 plates containing clones from neurula stage library (TNeu series, plate 49–50). In total, we have identified 20 pools, which altered the phosphorylation state of at least one of the signalling proteins in our screen (two examples are shown in Figure 5). We de-convoluted these pools and identified the active clone in each pool (Figure 5 and 6). We then sequenced each active clone, and confirmed that all of them were full-length (data not shown). Generally, we found that the gastrula library provided positive clones at a higher frequency (12 of 1152 clones, 1.04%) compared to clones derived from egg library (7 of 1536 clones, 0.46%). Therefore, it might be more efficient to perform subsequent functional screens using only the TGas and TNeu libraries. Of the 20 clones we identified, 4 had no previous known function (20%), while the remaining 16 did have previous functions described in *Xenopus* (Table 2). However, out of the 16 genes with previously known functions in *Xenopus*, only 4 had been shown to regulate signalling (clones not in bold in Table 3). Importantly, our data confirmed their known roles, which reinforced our confidence of the specificity of our method. Finally, of the 16 clones with known functions in *Xenopus*, most (15 out of 16) have orthologues with known function in other species, which suggests that the outcome of our screen may be applicable to other organisms.

**Table 2:** Classification of the positive clones identified in the screen.

*Clones with known functions in Xenopus*		
Classification	number	Percentage^a^
No known function	4	20%
Known function	16	80%

*Positive clones categorised according to their predicted or established functions* b		
Classification	number	Percentage^a^
Cell signalling	7	35%
Transcription	4	20%
Cell cycle/apoptosis	1	5%
Post-transcription/translation	1	5%
Other functions	7	35%
Functionality unknown	0	0%

*Positive clones categorised according to signalling pathways modulated* c		
Pathway	number	Percentage^a^
MAPK/Erk	11	55%
PI3K/Akt	5	25%
TGF-β/Nodal	3	15%
BMP	4	20%

Values were given as percentages against total clone numbers identified in the screen (*n* = 20).

a Percentage of clones having at least one publication describing their functions in *Xenopus*.

b 6 functional groups were established as described.

c A total of 20 positive clones have been identified. Clones that modulate the activities of more than one signalling pathways, are counted in each group.

**Table 3:** Summary of the positive clones identified in the screen.

GenBank ID	Clone ID	Gene name	Effect on signalling	Notes^a^
***A. Genes modulate Erk activity***				
**CT010561.2**	**TEgg009F05**	***fbxo43; F-box protein 43***	**activation, blastula**	
**CT030376.1**	**TEgg048a17**	***tmem209; transmembrane protein 209***	**activation, gastrula**	
**AL886760.2**	**TEgg048m10**	***gemin2; gem (nuclear organelle) associated protein 2***	**activation, gastrula**	
CR761362.2	TEgg071k05	*rhoa; ras homolog gene family, member A*	activation, gastrula	
**CT025377.2**	**TGas068f06**	***gnai1; guanine nucleotide binding protein (G protein), alpha inhibiting activity polypeptide 1***	**activation, gastrula**	
**CR761447.1**	**TGas103n06**	***foxh1; forkhead box H1***	**activation, gastrula**	
CR942555.2	TGas120g11	*cdc42; cell division cycle 42*	activation, gastrula	
**CR942588.2**	**TGas124n10**	***tbx6; T-box6***	**activation, gastrula**	
**CT025433.2**	**TGas120f24**	***fndc3a; fibronectin type III domain containing 3A***	**activation, gastrula**	
**CR761845.2**	**TGas135n07**	***eif3m; eukaryotic translation initiation factor 3, subunit M***	**activation, gastrula**	
**CR761503.2**	**TGas056c07**	***ppp1r8; protein phosphatase 1, regulatory subunit 8***	**inhibition, gastrula**	**Also inhibits Akt and BMP**
***B. Genes modulate PI3K/Akt activity***				
**CR761141.2**	**TEgg046d13**	***prkaca; protein kinase, cAMP-dependent, catalytic, alpha***	**inhibition, gastrula**	
**CR761314.2**	**TEgg049a02**	***rere; arginine-glutamic acid dipeptide (RE) repeats***	**inhibition, gastrula**	
**AL967388.2**	**TGas121j24**	***nxf1; nuclear RNA export factor 1***	**inhibition, gastrula**	
**CT030539.1**	**TGas122g01**	***ulk4; unc-51-like kinase 4***	**inhibition, gastrula**	
***C. Genes modulate BMP activity***				
CR760475.2	TNeu118d19	*wnt8a; wingless-type MMTV integration site family, member 8A*	inhibition, gastrula	
**CR760999.2**	**TEgg063n04**	***dpepe; dipeptidase E***	**inhibition, gastrula**	
**CR761501.2**	**TGas068o03**	***fezf2; FEZ family zinc finger 2***	**inhibition, gastrula**	**Also activates TGFβ/Nodal**
***D. Genes modulate TGF-β/Nodal activity***				
**AL782529.2**	**TGas079c23**	***ddx55; DEAD (Asp-Glu-Ala-Asp) box polypeptide 55***	**activation, gastrula**	
CR761456.2	TGas124h10	*nodal 1; nodal homolog 1*	activation, gastrula	

Clones that have not been previously reported in the literature for their roles in regulating signalling events are shown in **bold**.

a Notes describes additional effects on the activities of different signalling pathways.

Of the 20 clones, only one showed an effect at the blastula stage, while all the others did not affect signalling until the gastrula stages (Table 3). Although several clones induced changes in signalling activities at the neurula stage (data not shown), the changes seen in these clones were already present at the gastrula stage. These results suggest that assaying neurula stage embryos may not provide additional information that cannot be already gained by assaying the blastula and gastrula stages.

We next analysed the category of molecules we had identified during the screen. The largest group (7 clones, 35%) is comprised of signalling molecules, as expected (Table 2). However, a significant proportion of identified genes have predicted functions that are not normally associated directly with signalling, including genes that encode transcription factors (4 clones, 20%). This indicates that the screen did not only identify direct modulators of signalling, but also identified genes that are likely to act upstream of signalling (Table 2).

Finally, more than half (55%) of the molecules identified in the screen modulated Erk activity, while a quarter modulated Akt (25%), and 15% of clones modulated either BMP or TGF-β/Nodal signalling. Only two clones showed multiple activities (*ppp1r8* and *fezf2*, Table 3). Of the 11 clones identified able to modulate Erk phosphorylation levels during blastula or gastrula stages (Figure 6A), 2 (*rhoa*, and *cdc42*) had previously been shown to modulate Erk signalling [32], [33], thus providing further confirmation of the specificity of the screen. Of the remaining 9 clones, only one, *F-box protein 43* (*fbxo43*, also known as *erp1* or *emi2*), was able to induce a phosphorylation of Erk at the blastula stage. In *Xenopus* and mammalian models, Fbxo43 has been shown to mediate cytostatic arrest by inhibiting the anaphase-promoting complex (APC/C) [34]–[36]. Interestingly, the stability of Fbxo43 is regulated by a Mos-MEK-MAPK-p90RSK-dependant phosphorylation event, resulting in the inhibition of the APC/C complex and thus cytostatic arrest in metaphase II [37]. We report here that Fbxo43 is also able to activate MAPK/Erk signalling when mis-expressed. It would be interesting to investigate possible cross-regulation and feedback mechanisms between Fbxo43 and Erk. In addition to *rhoa* and *cdc42*, seven other clones lead to the hyper-activation of Erk signalling at the gastrula stage (Table 3). *Transmembrane protein 209* (*tmem209*), also known as *NET31*, is a nuclear envelope protein. Elevated level of *tmem209* promotes cell growth and human lung cancer [38]. Our results indicate that the oncogenic effect of Tmem209 might be due to its ability to activate the Erk pathway. *Gem associated protein 2* (*gemin2*), also known as *sip1*, encodes a zinc finger/homeodomain containing protein and is highly expressed during early neural development [39]. Gemin2 has been shown to interact with SMN (Survival of Motor Neuron) and Smad proteins and to regulate sequential neural fate decisions [40], [41]. In addition, *gemin2* has been associated with motor neuron diseases [42]. Gnail is a G protein inhibitory subunit [43]. As it is required for regulating cellular cAMP signalling cascades by directly binding to adenylyl cyclase, it is possible that changing its expression level would result in changes of MAPK/Erk activity [44]. Tbx6 is a transcription factor involved in mesoderm specification [45]. Knockdown experiments in *Xenopus* suggest that *tbx6* is important for the formation of paraxial mesoderm and neural crest differentiation [46], [47]. Overexpression of Tbx6 induces *fgf8* expression, which might explain why *tbx6* overexpression results in the activation of Erk [48]. Foxh1 (or Fast1) is a transcription factor, which binds to the Smad2/Smad4 complex, and is necessary for the expression of a subset of Nodal target genes [49]. However, our results indicate that mis-expression of *foxh1* also caused an increased in Erk phosphorylation, suggesting a possible crosstalk between Nodal and MAPK/Erk signalling pathways. Fndc3a is a fibronectin-related protein, which is required for the adhesion between spermatids and Sertoli cells in testis, and mutations in the *Fndc3a* gene result in mouse infertility [50]. *Eukaryotic translation initiation factor 3*, *subunit M* (*eif3m*) is a translation initiation factor that is highly expressed in human cancer cell lines and colon cancer tissues and *eif3m* knockdown impairs cell proliferation [51]. Only one gene, *protein phosphatase 1 regulatory subunit 8* (*ppp1r8*) was found to decrease Erk phosphorylation at stage 10.5. Ppp1r8 is an important regulatory subunit of the protein phosphatase 1 complex and its mis-expression could result in a global decrease in protein phosphorylation, including Erk [52].

We have also identified 5 clones that decreased Akt phosphorylation (Figure 6B and Table 3). These include *cAMP-dependent protein kinase catalytic subunit alpha* (*prkaca*), the alpha catalytic subunit of cAMP-dependent protein kinase (PKA). Prkaca has been reported to be a crucial regulator in meiotic and mitotic arrest [53]. The free monomeric alpha subunit is highly catalytically active when it is not associated with regulatory units, thus resulting in excessive PKA activation and subsequent cell cycle arrest [54], [55]. Another gene that inhibits Akt phosphorylation is *arginine-glutamic acid dipeptide* (*rere*) gene, a member of the atrophin family of arginine-glutamic acid (RE) dipeptide repeat-containing proteins. Over-expression of such families of proteins triggers apoptosis, cytotoxicity, and neurodegeneration [56]–[58]. The third gene in this group is *nuclear RNA export factor 1* (*nxf1*) gene, which belongs to a family of nuclear RNA export factor genes. Its protein product associates with NTF2-related export protein 1 to mediate transportation of nuclear mRNA into the cytoplasm [59]. The fourth gene in this group is *ulk4 unc-51-like kinase 4*, which is part of the ubiquitously expressed Ser/Thr-specific unc-51-like kinases family (ULKs). Mutations in the unc-51 gene family cause defects in axonal elongation and axonal structures in *C. elegans* [60]. In humans, single-nucleotide polymorphism in *ULK4* has been associated with multiple myeloma, however its molecular mechanism remains unclear [61]. Finally, *ppp1r8*, which we described previously as the sole inhibitor of Erk found in the screen, also led to a decrease in Akt phosphorylation.

The screen also identified three genes modulating the phosphorylation level of Smad1/5/8 (Figure 6C and Table 3). One of these genes is *Wingless-type MMTV integration site family, member 8A* (*wnt8a*), discussed previously. In addition, we discovered that overexpression of *FEZ family zinc finger 2* (*fezf2*) also decreases Smad1 phosphorylation. Fezf2 has been shown to play an important role in forebrain, diencephalon, and olfactory placode development [62]–[65]. In addition, *Fezf2* has been shown to regulate the differentiation of midbrain dopaminergic neurons, as well as axon projections between thalamus and cerebral cortex [66]–[68]. *dipeptidase E* (*dpepe* or *pepE*) is the last of this group. Dpepe retains strong sequence identity with bacterial PEPE gene, which cleaves N-terminal aspartyl peptides [69], [70].

Finally, we identified 3 genes that change the phosphorylation level of Smad2 (Figure 6D and Table 3). The first gene in this category was *Nodal-related 1* (*xnr1*), which had been identified for its ability in regulating left-right axis determination in *Xenopus* [71]. Nodal-related 1 belongs to the TGF-β superfamily and binds to TGF-β type I/II receptors to initiate signalling through Smad2/3 [72]. The second gene within this group is *DEAD (Asp-Glu-Ala-Asp) box polypeptide 55* (*ddx55*), a member of the DEAD box protein family characterized by the conserved motif Asp-Glu-Ala-Asp (DEAD) and a putative RNA helicases. The DEAD box protein family genes have been implicated in several cellular processes related to alteration of RNA secondary structure, and involved in diverse cellular functions including spermatogenesis, embryogenesis, cell growth, and division [73], [74]. The third clone in this category is *fezf2*, mentioned previously as an inhibitor of Smad1/5/8 phosphorylation. We found that *fezf2* overexpression led to an increase in Smad2 phosphorylation at gastrula stage. Notably, changes on the phosphorylation level of Smad1/5/8 and Smad2 caused by *fezf2* mis-expression was similar to that caused by *wnt8a* mis-expression, suggesting that Fezf2 may promote canonical Wnt signalling.

### Expression pattern of the clones isolated during the screen

**Figure 7:**
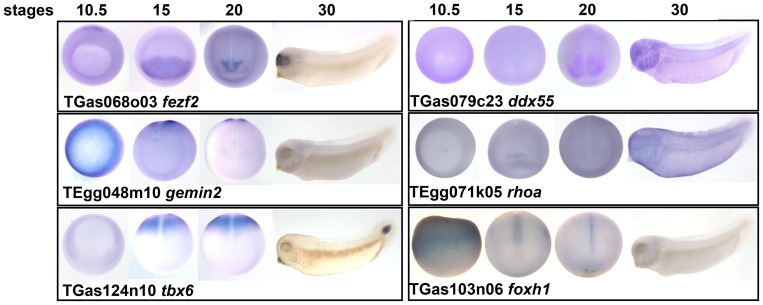
Whole-mount *in situ* hybridisation images on clones with regionalised expression patterns. For each clone the corresponding clone number and *Xenopus* gene symbol are shown. Vegetal view (stage 10.5 except for *foxh1*, which is side view); dorsal view (stage 15 and 20, posterior is up); lateral view (stage 30, anterior is to the left).

We next endeavoured to ascertain the spatial expression patterns of the genes we identified by whole mount *in situ* hybridisation. Half the Erk regulators (5 out of 11) and all of the BMP (3 out 3) and Nodal regulators (2 out 2) had regionalised expression patterns (Table 3 and Figure 7). However, none of the Akt inhibitors isolated during the screen showed localised expression patterns (data not shown). For the Erk regulators, *fbxo43* had been previously shown to be expressed in the mesoderm at gastrula stage [75]. *rhoa* expression was slightly enriched in the anterior most region at stage 15. The expression of *gemin2* was localised to the mesoderm of gastrula embryos and then in the closing blastopore at stage 15 and 20 (Figure 7). *tbx6* mRNA was localised in the posterior paraxial mesoderm at the gastrula and neurula stages, and in the tail at the tailbud stage (Figure 7) [47]. *foxh1* was enriched in the dorsal midline at the neurula stages (Figure 7). Consistent with this finding, others have reported that the *X. laevis foxh1* orthologue is expressed in the notochord at the tailbud stage [76].

For TGF-β regulators, the expression patterns of *xnr1* and *wnt8a* had been previously described [77]–[79]. *fezf2* was expressed in the anterior neural plate and later is the forebrain, similar to what had been reported in zebrafish and mice (Figure 7) [63], [80]. Finally, we found that *ddx55* was expressed in the head and branchial arches at tadpole stage (Figure 7), similar to previously reported [81].

In conclusion, we have successfully identified a number of genes for their novel roles in regulation of signalling pathways during early embryogenesis. By combining data from our over-expression screen, regional and temporal expression profiles, and additional knockdown experiments, it will be possible to establish the mechanism of these regulators and their roles in early *Xenopus* embryogenesis. Since signal transduction pathways are highly conserved amongst vertebrates, it is likely that our findings will have implications to our understanding of the molecular mechanisms that regulate signalling in other organisms. Interestingly, although all the genes identified in this screen had been previously identified and studied at some level, the majority had not previously been implicated in regulating the activity of signalling pathways. Finally, with another approximately 7000 EST clones to be screened, it is clear that there are many more regulators that signal transduction pathways which remain to be uncovered using this strategy.

## Supporting Information

Figure S1 **Flowchart of pilot screen.** 12 clones with known activities have been used during the pilot screen. The corresponding position of each clone was located in the EST library (black dots) together with the seven clones of the same column (grey dots). After that, the whole column (black and grey dots) were re-arrayed into one column of a new 96-well plate. Bacteria containing different clones were cultured individually and pooled together for subsequent plasmid extraction, linearization, and mRNA transcription to achieve 12 mRNA pools each containing 8 clones. mRNA pools were injected into *X. laevis* embryos at 1–2 cell stage and collected at specific stages as described in the main text. Collected embryos were homogenised and their protein contents extracted for subsequent Western blot analyses.
(TIF)
