# Supplementary figures and images for: A Functional Genome-Wide In Vivo Screen Identifies New Regulators of Signalling Pathways during Early Xenopus Embryogenesis
Source: PLoS One. 2013 Nov 14;8(11):e79469. doi: 10.1371/journal.pone.0079469 (PMC3828355; doi:10.1371/journal.pone.0079469)

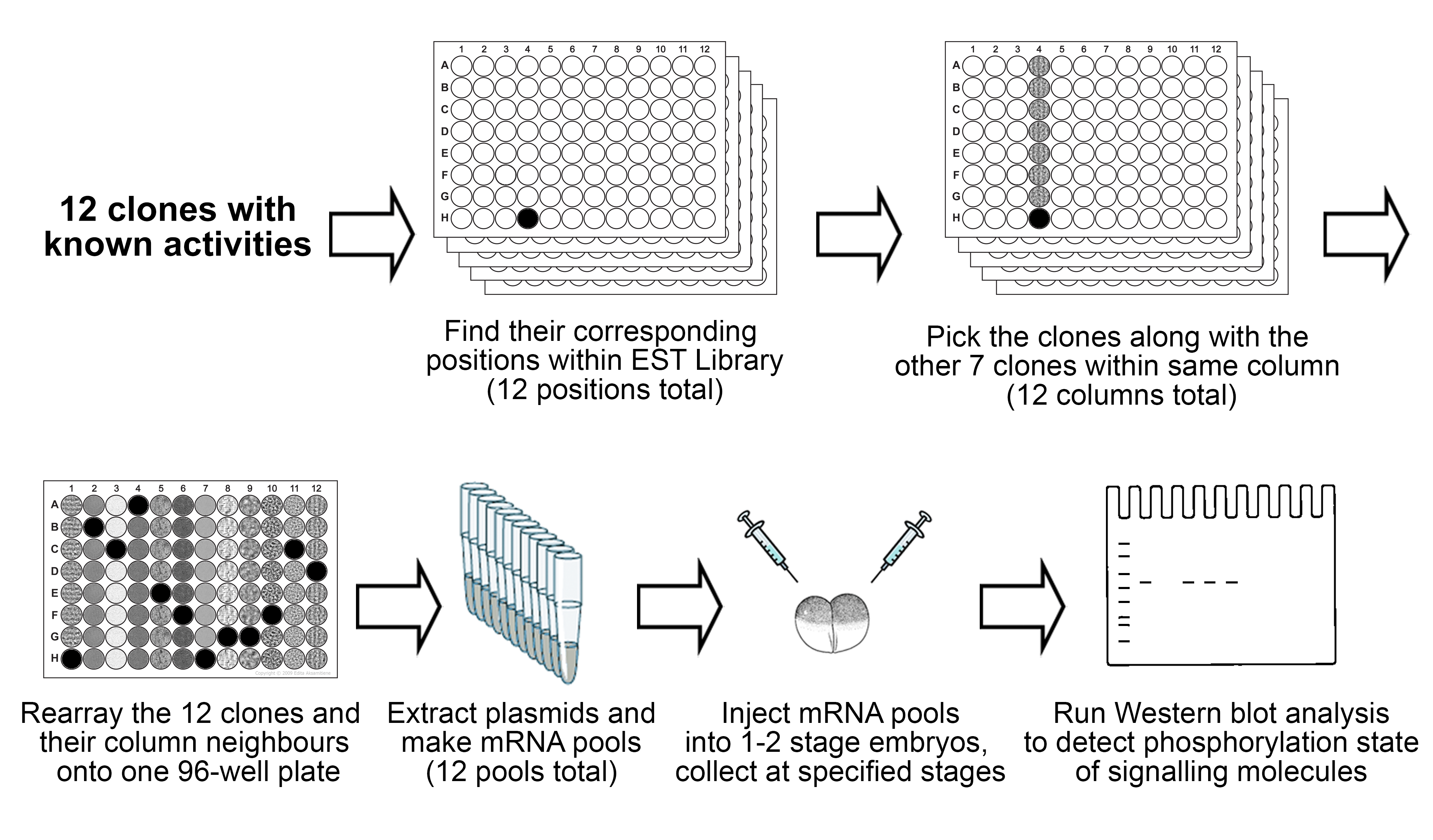

Supplement: Figure S1 — Flowchart of pilot screen. 12 clones with known activities have been used during the pilot screen. The corresponding position of each clone was located in the EST library (black dots) together with the seven clones of the same column (grey dots). After that, the whole column (black and grey dots) were re-arrayed into one column of a new 96-well plate. Bacteria containing different clones were cultured individually and pooled together for subsequent plasmid extraction, linearization, and mRNA transcription to achieve 12 mRNA pools each containing 8 clones. mRNA pools were injected into X. laevis embryos at 1–2 cell stage and collected at specific stages as described in the main text. Collected embryos were homogenised and their protein contents extracted for subsequent Western blot analyses. (TIF) [file pone.0079469.s001.tif]
